# A Human "Brute"

**Published:** 1889-02-02

**Authors:** 


### A HUMAN "BRUTE."

Medical science, in its largest sense, has much to teach both moralists and legists. Human creatures, when they have attained a sufficient age, are justly held responsible for their actions both in law and in social life. But the degree of responsibility in different persons varies almost infinitely, and no class of men are so well able to understand and account for these varying degrees of responsibility as medical men of philosophic mind. In a lecture recently delivered at the Royal College of Surgeons, London, Mr. Jonathan Hutchinson recalled to the memory of his hearers the skeleton of a dwarf which is the property of the Norwich Museum. This skeleton has limbs which in certain important particulars more nearly resemble those of an animal than those of a man. The humerus and femur—that is, the long bones of the arm and leg—are like those of a bear. The collar bones are joined to the breast bone in an entirely different way from what is usual, and seem as if they were intended to move more or less freely on the articulating surfaces. The appearance of the whole skeleton is most striking and peculiar. The head, however, which is generally held to give the most significant indications of character, is not unusually small, although it is ill-formed and narrow. This man, who was so brute-like in his limbs, appears to have corresponded in charactor to his physical formation. He attempted to murder both his wife and child by poison, and was executed for his crime. He displayed an utter want of anything like human understanding or feeling. He was entirely callous to the last, showing neither fear nor remorse. The peculiarity in this case was that the characteristics of an animal nature showed themselves so much more markedly in the limbs than in the head. This skeleton well illustrates a whole series of cases in which there seems in human beings a falling below the typical human level, and the manifestation of characteristics proper to the lower animals. It is impossible to deny the kinship between human beings and the lower animals; and it is very necessary to constantly recognise the fact for the sake of both animals and men.

